# Thermoplastic Polyurethane-poly(*N*-isopropylacrylamide) Copolymer for Selective Uptake of Alcohol from Aqueous Solution

**DOI:** 10.3390/ma17122795

**Published:** 2024-06-07

**Authors:** Fei Wang, Tiexin Cheng, Guangdong Zhou

**Affiliations:** College of Chemistry, Jilin University, Changchun 130061, China; wangfei21@mails.jlu.edu.cn (F.W.); chengtx@jlu.edu.cn (T.C.)

**Keywords:** thermoplastic polyurethane, poly(*n*-isopropylacrylamide), selective uptake

## Abstract

Ethanol possesses high economic value, but as an industrial waste, it poses harm to human health and the environment. The paper describes the preparation of a thermoplastic polyurethane-poly(*n*-isopropylacrylamide) (TPU-PNIPAM) copolymer designed to selectively uptake alcohol in aqueous solution. The material was created by bonding TPU and PNIPAM together through intermolecular hydrogen bonds, enhancing its hydrophobic properties and making it easier to interact with alcohol molecules. As the amount of PNIPAM in TPU increases, the number of hydrophobic isopropyl groups in TPU-PNIPAM also increases, leading to an enhanced selective uptake ability of TPU-PNIPAM for alcohols in aqueous solution. When the temperature reaches 55 °C, the hydrophobic groups in TPU-PNIPAM are more exposed, further enhancing the selective uptake ability of TPU-PNIPAM for alcohols in aqueous solution. TPU-PNIPAM demonstrates selective preferential uptake for various concentrations and types of alcohol in aqueous solutions. The material’s selective uptake performance for alcohols increases with their hydrophobicity, so TPU-PNIPAM exhibited the best adsorption performance for a 10 wt% n-propanol solution under the combined effect of steric hindrance. In addition, TPU-PNIPAM exhibited selective adsorption for other organic solvents, which demonstrated the universality of TPU-PNIPAM in removing contaminants from aqueous solutions.

## 1. Introduction

Bioethanol is a promising clean liquid fuel that could significantly reduce CO_2_ emissions if used instead of gasoline [1]. In addition to its economic value, ethanol is an industrial pollutant that has adverse effects on both the environment and human health [2,3]. Since ethanol molecules have similar molecular sizes and dipole moments as water molecules, removing ethanol from aqueous solutions is very challenging [4]. Especially for dilute ethanol/water mixtures, this implies the need to selectively trap small amounts of ethanol in solution and reject water molecules [5]. Therefore, there is interest in developing a type of material that can selectively uptake ethanol. By preparing a hydrophobic film with specific properties, ethanol can be separated from aqueous solutions using the pervaporation technique [6,7,8]. However, the pervaporation process has high requirements for experimental equipment, and the process is complex [9,10,11,12,13]. By contrast, removing ethanol from water through simple adsorption is more energy efficient, environmentally friendly, and economically competitive [14,15]. Various materials, such as silica molecular sieves [16], metal–organic frameworks (MOFs) [17,18], polymer resins [19], and carbon nanotubes [4], have been utilized as materials for ethanol uptake. To achieve selective uptake of ethanol, it is important to consider London dispersion interactions. G. F. de Lima et al. constructed a water-stable MOF named Zn_2_(BDC)_2_(TED), and the preferential uptake of ethanol over water in the framework was attributed to London dispersion interactions between the ethanol alkyl group and the benzene ring present in Zn_2_(BDC)_2_(TED) [20]. By masking or removing the hydrophilic group, the hydrophobic region can be enlarged, allowing for more accurate separation of water and alcohol molecules [21]. The most effective uptake effect can be achieved when matching the length of the hydrophobic chain with that of the alcohol chain through dispersion interactions between the alkyl groups [20].

Thermoplastic polyurethane (TPU) is a copolymer containing both soft and hard segments [22,23]. The soft segment consists of a long, low polymer chain that provides hydrophobic alkyl sites. The hard segments, on the other hand, consist of polar groups like urethane groups, which offer abundant hydrophilic hydrogen-bonding sites. Due to these abundant hydrogen-bonding sites, the target materials can be introduced into the TPU material through in situ polymerization, template leaching, or thermally induced phase separation to obtain composites with specific properties [24]. José et al. uniformly dispersed multi-walled carbon nanotubes in TPU, and the resulting composite material had a more complete conductive network conducive to electron conduction [25]. Solmaz et al. doped polyvinyltrimethoxysilane (P-VTMS) with TPU to obtain three-dimensional aerogel composites with thermal insulation properties and a special porous structure, which could be used as thermal insulation materials [26]. Liu et al. mixed a hybrid flame retardant (ATi_3_C_2_T_x_) synthesized from titanium carbide (Ti_3_C_2_T_x_) and aluminum hypophosphate (AP) into TPU, and the obtained composite material had good fire resistance [27]. Poly(N-isoproplacrylamide) (PNIPAM) contains both hydrophilic amide groups (-CONH-) and hydrophobic isopropyl groups (-CH(CH_3_)_2_-), exhibiting both hydrophilic and hydrophobic properties [28,29]. 1H MAS NMR quantitatively characterized the preferential alignment of alcohol molecules near PNIPAM in ethanol/water mixtures, independent of temperature [30]. Meanwhile, as the temperature increases, PNIPAM becomes more hydrophobic, making it more effective in adsorbing alcohols. However, its poor mechanical properties limit its practical application. Composite polymers of PNIPAM with three-dimensional polymeric network structures can be constructed by incorporating crosslinking agents to enhance their mechanical properties. Ou R. et al. have successfully prepared TPU-PNIPAM composite membranes that possessed the ability to separate both oil-in-water and water-in-oil emulsions [31]. Hydrogen bonds were formed between the poly-(ethyl carbamate) group and the acrylamide group, which made it possible for PNIPAM to be evenly coated on the surface of TPU. As the amount of PNIPAM increased, the number of hydrophilic sites in TPU and PNIPAM decreased, while the surface roughness increased. This led to the hypothesis that PNIPAM can be used to flexibly modify TPU and act as a host–guest switch for ethanol/water selective uptake.

This article describes the loading of PNIPAM onto the surface of TPU to prepare a type of TPU-PNIPAM copolymer and also explores the selective and preferential uptake properties of the TPU-PNIPAM copolymer for ethanol molecules in aqueous solution as well as the effects of various conditions on the properties of the copolymer.

## 2. Materials and Methods

### 2.1. Materials

Thermoplastic polyurethane pellets (Zhongcheng Plastic Technology Co., Ltd., Guangzhou, China), tetrahydrofuran, sodium chloride, isopropanol (Sinopharm Chemical Reagent Co., Ltd., Yingkou, China), *N*-isopropylacrylamide (Shanghai McLean Biochemical Technology Co., Ltd., Shanghai, China), N,N-methylenebisacrylamide (Anhui Zaisheng Technology Co., Ltd., Ma’anshan, China), ammonium persulfate(Anhui Sunrise Technology Co., Ltd., Ma’anshan, China), anhydrous ethanol (Shanghai Aladdin Biochemical Technology Co., Ltd., Shanghai, China), anhydrous methanol (Beijing Chemical Plant, Beijing, China), propanol (Tianjin Yongda Chemical Reagent Co., Ltd., Tianjin, China), and distilled water; the above reagents were analytically pure and were used directly without further purification.

### 2.2. Preparation of TPU-PNIPAM

The TPU particles were dried in an oven for 12 h prior to the experiments. Then, 2 g TPU was weighed and dissolved in 8 mL tetrahydrofuran, heated, and stirred until it became homogeneous and transparent. Pre-ground sodium chloride particles were then added and mixed thoroughly. The mixture was swiftly poured into cylindrical molds that had a diameter of 30 mm and a depth of 10 mm. After being left in a fume hood for 24 h, the molds were transferred to an oven where the mixture was dried at a temperature of 55 °C. After thoroughly drying and forming the product, it was removed from the mold and sanded. Then, it was placed in distilled water and the water was replaced once every 8 h until no sodium chloride precipitated. Once this was achieved, the reshaped TPU material (1.5 g) was dried and stored for future use.

Quantities of 0.50 g, 1.00 g, and 1.50 g of N-isopropyl acrylamide were weighed to use as the polymerization monomer. Additionally, 1% of the monomer mass of N,N-methylenebisacrylamide was also weighed and added as a crosslinking agent. The mixture was stirred in 30 mL of distilled water until dissolved, creating a colorless transparent solution. The TPU material was added to the reaction solution, to which 100 μL of 10 wt% ammonium persulfate solution was added as the initiator. The reaction was carried out in an oxygen-free environment at 70 °C for 3 h. After the reaction, the product was washed with distilled water at 25 °C and 45 °C to remove any unreacted monomer. The TPU-PNIPAM copolymer was then dried to obtain T-N_0.5_, T-N_1.0_, and T-N_1.5_.

### 2.3. Characterization of Materials

The sample was characterized using a Quattro ESEM scanning electron microscope (Thermo Scientific, Waltham, MA, USA). The sample was fixed onto the sample table with conductive adhesive and coated with a thin layer of gold to prevent electrostatic charging.

The sample was analyzed using a Nicolet IS5 Fourier infrared spectrometer (Thermo Scientific, Waltham, MA, USA). The product was first cut into a fine powder, then mixed thoroughly with potassium bromide at a ratio of 1:100. After that, the mixture was pressed into thin slices using a tablet press. Finally, it was scanned 32 times within the wavelength range of 4000–400 cm^−1^.

Raman spectrum analysis was conducted using a LabRAM HR Evolution high-resolution laser Raman spectrometer (HORIBA France SAS, Paris, France) with a laser wavelength of 514 nm and scanning range of 800–1800 cm^−1^.

A TGA/SDTA851 thermogravimetric analyzer (METTLER TOLEDO, Zurich, Switzerland) was used to perform thermogravimetric analysis of the experimental samples in a N_2_ atmosphere. The temperature was increased from room temperature to 700 °C at a rate of 20 °C/min.

### 2.4. Physical Property Test

The tensile properties of the samples were tested using a CMT-20 electronic universal testing machine (Jinan Liangong Testing Technology Co., Ltd., Jinan, China) at a rate of 150 mm/min.

### 2.5. Maximum Uptake Capacity Test

The equation of maximal absorption capacity (*Q_m_*) is expressed as follows:

(1) $$Q_{m}=\frac{m_{t}-m_{0}}{m_{0}}\times 100\%$$

where *m*_0_ and *m_t_* are the mass of the monolith before and after the experiment, respectively.

### 2.6. Uptake Performance Test

The effectiveness of TPU-PNIPAM in selectively adsorbing alcohol/water mixtures was tested using a SHIMADZU GC-8A gas chromatograph with a Q column (Shimadzu, Kyoto, Japan). High-purity helium served as the carrier gas and the pre-column pressure was maintained at 400 kPa, the injection column pressure at 220 kPa, and the reference column pressure at 230 kPa. The helium flow rate was set at 37.5 mL/min. The current flowing through the bridge was measured at 140 mA. The injection and detection chambers were maintained at a temperature of 200 °C, while the column box was heated to 180 °C. A single injection volume of 0.2 μL was used for each test. In a closed bottle, 3 mL of an alcohol/water mixture was added and the prepared TPU-PNIPAM material was placed inside the bottle for each test. To avoid the influence of ethanol volatilization on the solution concentration during sampling, the concentration change of the solution without uptake material was also measured. Samples were collected and documented at 10 min intervals to measure the alteration in the concentration of the solution within a 90 min timeframe. During the alcohol concentration change test, *c*_0_ represents the initial concentration of the alcohol solution, while *c_t_* denotes the concentration of the alcohol solution at time *t*.

### 2.7. Standard Deviation and Statistical Analysis

All experiments were repeated three times, and the mean value and standard deviation were calculated based on Equations (2) and (3), as follows:

(2) $$\bar{x}=\frac{\sum_{i=1}^{n}x_{i}}{n}$$

(3) $$s=\sqrt{\frac{1}{n-1}\sum_{i=1}^{n}{\left(x_{i}-\bar{x}\right)}^{2}}$$

where $x_{i}$ represents a single measurement, *n* represents the number of experimental replicates, $\bar{x}$ represents the mean, and *s* represents the standard deviation.

## 3. Results and Discussion

### 3.1. Characterization Analysis

#### 3.1.1. Morphology Analysis

**Figure 1:**
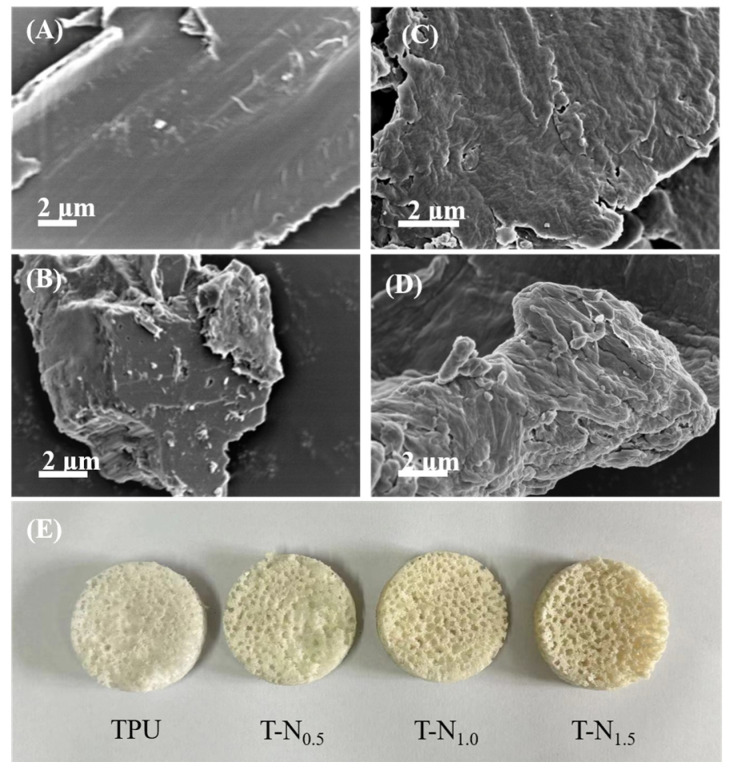
SEM images of (**A**) TPU, (**B**) T-N_0.5_, (**C**) T-N_1.0_, and (**D**) T-N_1.5_. (**E**) The appearances of TPU, T-N_0.5_, T-N_1.0_, and T-N_1.5._

As per Figure 1A, it can be observed that the surface of TPU had a smooth layer-like structure. T-N_0.5_, T-N_1.0_, and T-N_1.5_ exhibited the characteristics of stacked layers (which can be seen in Figure 1B–D). As the mass of the polymerized monomers increased, the density of the stacks also increased, leading to a rougher surface. Figure 1E illustrates that TPU was white. As the PNIPAM loading increased, the color of the copolymer gradually deepened and became light yellow. In conclusion, this demonstrated that PNIPAM was successfully loaded onto the surface of TPU through in situ polymerization.

#### 3.1.2. FT-IR

**Figure 2:**
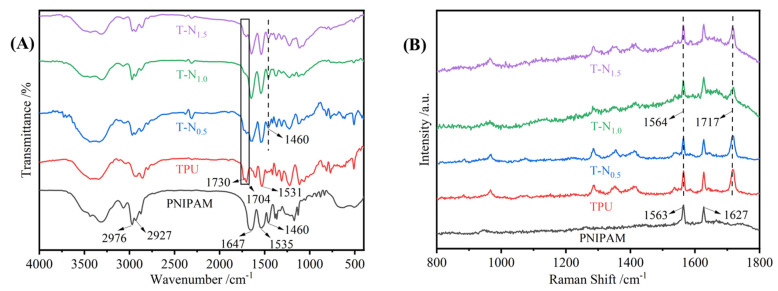
(**A**) FT-IR spectra of PNIPAM, TPU, T-N_0.5_, T-N_1.0_, and T-N_1.5_. (**B**) Raman spectra of PNIPAM, TPU, T-N_0.5_, T-N_1.0_, and T-N_1.5_.

In Figure 2A, the vibrational peaks observed at 2976, 2927, and 1460 cm^−1^ were ascribed to PNIPAM’s methyl (-CH_3_), methylene (-CH_2_-), and isopropyl (-CH(CH_3_)_2_) stretching vibrations. Two typical signals for amides were bands at 1647 cm^−1^ and 1535 cm^−1^. The former was assigned to the stretching vibration of C=O, and the latter was assigned to the bending of N-H and the stretching of C-N [32]. The TPU spectrum exhibited vibrational peaks at 1730 and 1704 cm^−1^, which were attributed to the free carbonyl and hydrogen-bonded carbonyl stretching vibrations, respectively. The amide absorption peak caused by coupling of the bending vibration of the N-H bond with the tensile vibration of the C-N bond was observed at 1531 cm^−1^ [33]. The characteristic peaks of TPU and PNIPAM were observed simultaneously in the spectra of T-N_0.5_, T-N_1.0_, and T-N_1.5_. The stretching vibrational peak of the carbonyl group in TPU was attributed to the vibrational peaks at 1730 and 1704 cm^−1^, while the isopropyl group in PNIPAM was responsible for the vibrational peak at 1460 cm^−1^. In comparison to that of TPU, the T-N_0.5_ spectrum demonstrated a significant reduction in the free carbonyl peak at 1730 cm^−1^. Furthermore, the peak was no longer observable in the spectra of T-N_1.0_ and T-N_1.5_, indicating the formation of a hydrogen bond between the carbonyl group and PNIPAM. The free carbonyl group transformed into a hydrogen-bonded carbonyl group, and the bonding between PNIPAM and TPU was achieved through hydrogen bonding.

#### 3.1.3. Raman

High-resolution laser Raman spectroscopy is used as a complementary method to FT-IR. In Figure 2B, the peaks observed at 1563 and 1627 cm^−1^ in the PNIPAM spectrum were attributed to the N-H deformation and C-N stretching vibrations in amides, while the peaks seen at 1717 and 1564 cm^−1^ in the TPU spectrum were attributed to the stretching vibrations of carbonyl and amide groups [34]. For TPU-PNIPAM, the carbonyl vibrational peak at 1717 cm^−1^ was slightly distorted and weakened. This could be explained by the formation of intermolecular hydrogen bonds between TPU and PNIPAM, which was consistent with the FT-IR spectral analysis.

**Figure 3:**
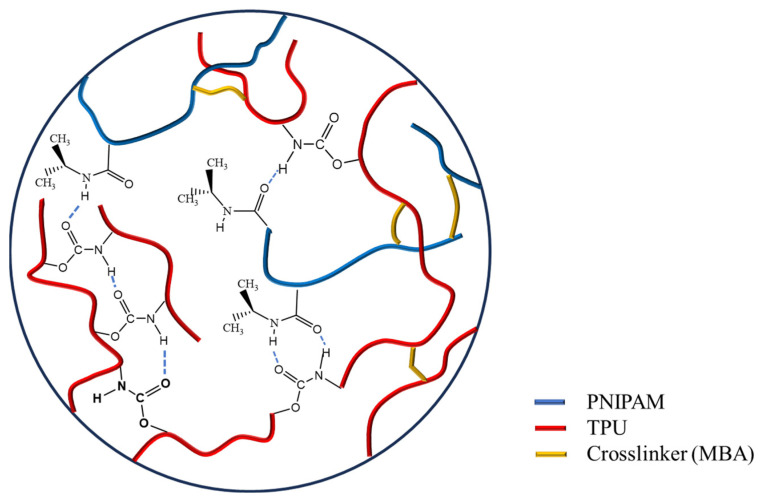
Hydrogen bonding mode in TPU-PNIPAM copolymer. The types of hydrogen bonds that may be formed in the copolymer are represented by the blue dashed line.

According to the results presented in Table 1, the isopropyl peaks of T-N_0.5_, T-N_1.0_, and T-N_1.5_ had relative areas of 2.16%, 2.93%, and 3.37% in the FT-IR spectra, respectively. The relative areas of the amino peaks of T-N_0.5_, T-N_1.0_, and T-N_1.5_ were 9.44%, 8.93%, and 7.24% in the Raman spectra, respectively. The spectroscopic characterization results obtained from FT-IR and Raman analyses indicated that PNIPAM was successfully loaded on the TPU surface via in situ polymerization. The bonding between the two occurred through the formation of an O-H hydrogen bond between the carbonyl oxygen and amino hydrogen. Due to the formation of hydrogen bonds between TPU and PNIPAM, the carbonyl and amino groups on the surface of both were heavily masked. These groups can form hydrogen bonds with water but were now covered. On the other hand, the hydrophobic isopropyl groups were exposed. This outcome inevitably led to an improvement in dispersion interactions between isopropyl and alcohol alkyl groups, and this improvement favored the selective uptake of alcohols. In other words, the hydrogen bonding between PNIPAM and TPU effectively enhanced the hydrophobicity of the TPU-PNIPAM copolymer, which was beneficial for the selective uptake of alcohol/aqueous solutions, as shown in Figure 3.

#### 3.1.4. TG

**Figure 4:**
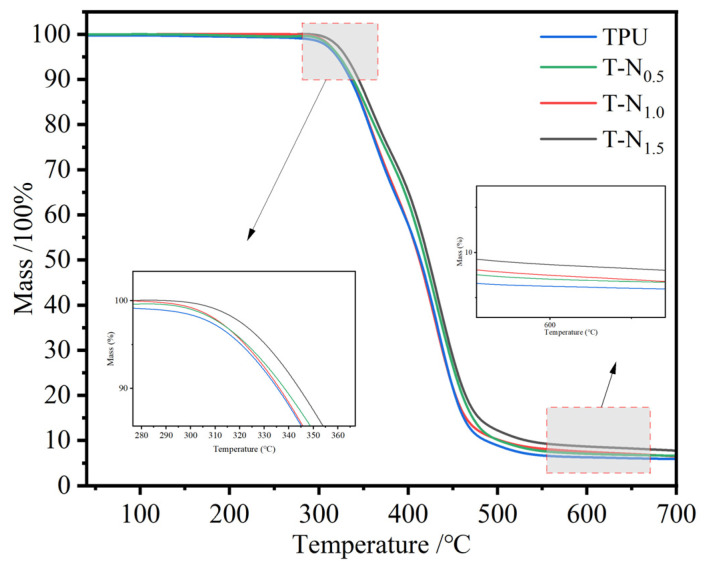
TG spectra of TPU, T-N_0.5_, T-N_1.0_, and T-N_1.5_.

The temperatures at which TPU and PNIPAM decompose are 310 °C and 350 °C, respectively [35,36]. Figure 4 displays the thermogravimetric curve of TPU-PNIPAM. The decrease in mass before decomposition was due to the evaporation of water that was adsorbed on the surface of the sample with the interstitial space. As the amount of PNIPAM on TPU increased, the loss of adsorbed water mass decreased progressively. As the number of hydrogen bonds between TPU-PNIPAM increased, there were fewer bonding sites left for water. This resulted in a decrease in the degree of uptake of airborne water. In other words, loading PNIPAM could effectively improve the hydrophobicity of TPU. The thermogravimetric curves of TPU-PNIPAM showed minimal changes compared to TPU due to the small mass loading of PNIPAM. At a thermal decomposition temperature of 650 °C, the masses of TPU, T-N_0.5_, T-N_1.0_, and T-N_1.5_ became 5.91%, 6.46%, 6.62%, and 7.74% of their original values, respectively.

### 3.2. Physical Property Test

**Figure 5:**
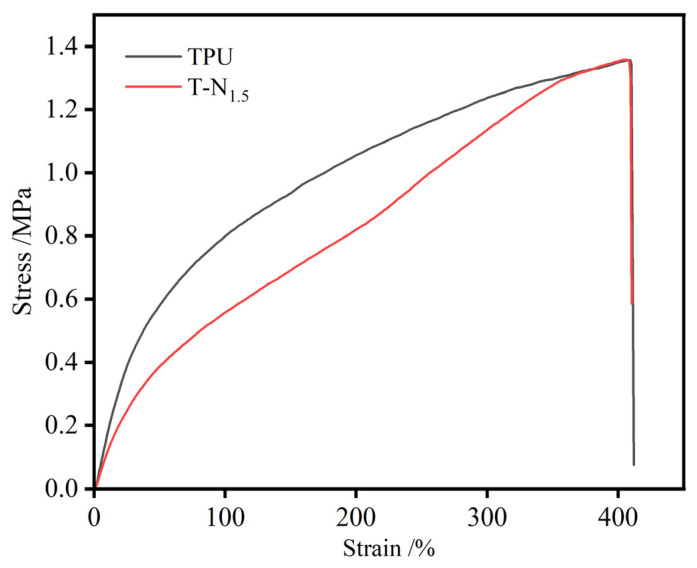
Tensile test curves of TPU and T-N_1.5_.

Based on Figure 5, it was clear that the loading of PNIPAM had no significant effect on the tensile properties of TPU. When the tensile fracture stress of TPU and T-N_1.5_ reached 1.3 MPa, the elongation at break reached over 410%, demonstrating that the material had good tensile properties.

### 3.3. Uptake Property Analysis

#### 3.3.1. Maximum Uptake Capacity

**Figure 6:**
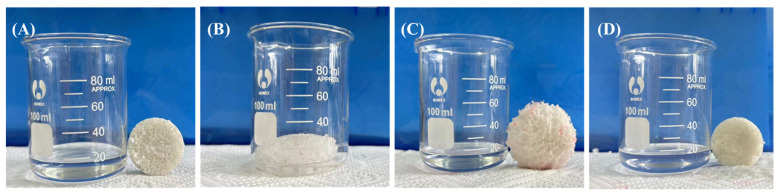
The behavior of the copolymer before (**A**), during (**B**), and after (**C**) uptake, and (**D**) its behavior after ethanol removal by extrusion.

The TPU, T-N_0.5_, T-N_1.0_, and T-N_1.5_ samples were immersed in ethanol for fixed time intervals, followed by removal and weighing to establish uptake equilibrium curves. Upon reaching saturation uptake, the ethanol absorbed by the material was extracted through simple extrusion before subjecting it to further rounds of ethanol uptake for a total of six cycles. As shown in Figure 6, the adsorbed copolymer underwent a significant volume increase. Once the adsorbed ethanol was extruded, the copolymer returned to its initial size.

**Figure 7:**
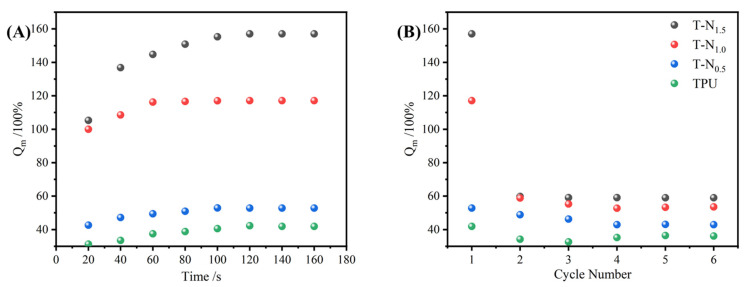
(**A**) The maximal uptake curves of TPU, T-N_0.5_, T-N_1.0_, and T-N_1.5_ for ethanol. (**B**) Repeated uptake properties of TPU, T-N_0.5_, T-N_1.0_, and T-N_1.5_ for ethanol.

The $Q_{m}$ of the copolymer was calculated using Equation (1). As depicted in Figure 7, TPU, T-N_0.5_, T-N_1.0_, and T-N_1.5_ exhibited ethanol uptake capabilities and achieved equilibrium within approximately 2 min. Moreover, an increase in PNIPAM loading on TPU led to enhanced maximal uptake capacity of the copolymer for ethanol. Notably, the maximal uptake capacity of T-N_1.5_ for ethanol reached 157.02%, which was approximately fourfold higher than that of pure TPU. After the initial uptake, the foam exhibited a decrease in its maximal uptake capacity, which eventually reached a stable state. Following six cycles of uptake–desorption, the maximal uptake capacities of T-N_1.5_, T-N_1.0_, T-N_0.5_, and TPU decreased from 157.02%, 117.09%, 52.84%, and 41.85% to 59.00%, 53.54%, 42.86%, and 36.11%, respectively. This was attributed to the presence of PNIPAM on the surface of TPU, which facilitated interaction between isopropyl groups and ethanol alkyl groups. The incorporation of more polymer enhanced its ability to interact with ethanol. However, conventional extrusion methods were unable to eliminate the adsorbed ethanol and may have resulted in polymer elution, consequently reducing the maximal uptake capacity in subsequent cycles.

#### 3.3.2. The Impact of PNIPAM Loading on the Uptake Performance

**Figure 8:**
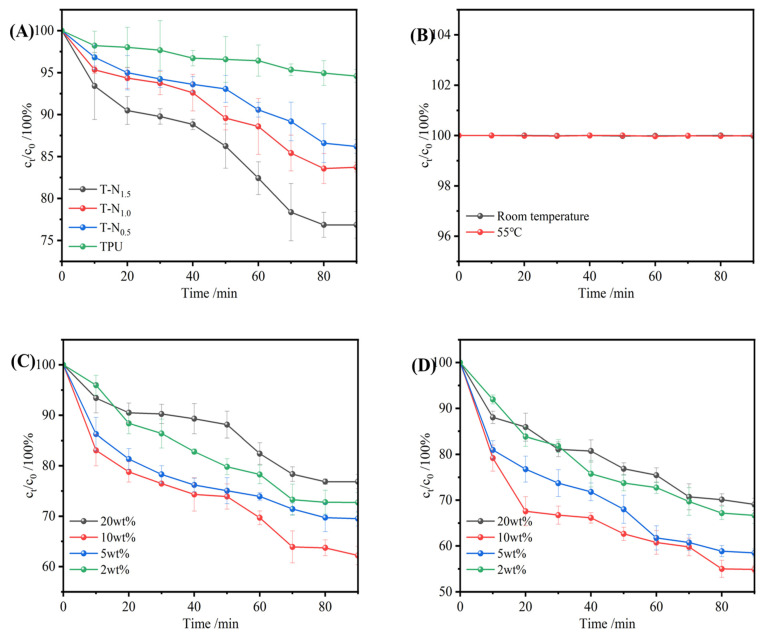
(**A**) Uptake of TPU, T-N_0.5_, T-N_1.0_, and T-N_1.5_ of 20 wt% ethanol solution. (**B**) Controlled experiment of 20 wt% ethanol solution at room temperature and 55 °C. (**C**) Uptake effect of T-N_1.5_ on different concentrations of ethanol at room temperature. (**D**) Uptake effect of T-N_1.5_ on different concentrations of ethanol at 55 °C.

In Figure 8A, it is demonstrated that the uptake of TPU resulted in a reduction of the concentration of the ethanol/water mixture solution by approximately 5%. This indicated that the TPU material displayed preferential uptake properties for ethanol. Moreover, the selective uptake of ethanol by TPU-PNIPAM was observed to increase with the input of polymerization monomers. After 90 min, T-N_0.5_, T-N_1.0_, and T-N_1.5_ uptake caused a decrease in the concentration of ethanol/water mixture solution by 13.8%, 16.3%, and 23.2%, respectively. This suggested that the selective uptake of the TPU-PNIPAM copolymer for the ethanol/water mixture solution was improved with an increase in PNIPAM loading on the TPU surface. As the concentration of the polymerized monomer NIPAM increased, the number of hydrogen bonds between PNIPAM and TPU also increased. This was because the carbonyl and amino groups, which can form hydrogen bonds with water molecules, were concealed, while a significant number of exposed isopropyl groups created London dispersion interactions with the ethyl group of ethanol. T-N_1.5_ was found to have the strongest selective uptake capacity for ethanol; its selective uptake capacity for ethanol within 90 min was about five times that of TPU. We could conclude that once the loading capacity of PNIPAM reached a certain value, the TPU-PNIPAM copolymer could effectively eliminate ethanol from an aqueous solution.

T-N_1.5_ was used as the uptake material in all subsequent experiments. The solution without any additional uptake material was tested to measure the concentration change every 10 min. This was done to exclude the effects of the solution’s own volatilization on the concentration. The results are depicted in Figure 8B. Under the conditions of this experiment, the concentration change that occurred due to ethanol volatilization was insignificant.

#### 3.3.3. Effect of Initial Ethanol Concentration on Uptake Performance

Ethanol solutions with 20 wt%, 10 wt%, 5 wt%, and 2 wt% mass fractions were used to investigate the effect of the initial ethanol/water concentration on the TPU-PNIPAM copolymer ‘s selective uptake performance. Figure 8C presents the results. TPU-PNIPAM exhibited selective uptake for varying concentrations of ethanol solutions. The best uptake performance was observed for the 10 wt% ethanol solution, which was reduced to 62.2% of its original concentration in 90 min, and the concentrations of 20 wt%, 5 wt%, and 2 wt% ethanol solution were decreased to 76.8%, 69.5%, and 72.7%, respectively. The TPU-PNIPAM material exhibited consistent uptake behavior for various concentrations of ethanol and water solutions. Moreover, the ethanol concentration in the solution decreased, indicating that TPU-PNIPAM had better uptake capacity for ethanol/water mixed solutions than for water alone. This proved that it selectively adsorbed ethanol.

The ethanol/water uptake curves were observed for various initial concentrations. The results showed that there was a rapid ethanol uptake phase in the first 20 min, followed by a smooth uptake phase between 20 and 50 min. Then, there was another rapid uptake phase from 50 to 70 min, and finally, the curve reached equilibrium with smooth uptake from 70 to 90 min. TPU-PNIPAM possessed hydrophobic properties that enabled it to adsorb ethanol selectively. During the first 20 min of the process, the uptake behavior was governed by kinetic diffusion, allowing ethanol to be quickly absorbed and arranged on the surface of TPU-PNIPAM, leading to a rapid decrease in the ethanol concentration in the ethanol/aqueous solution. When ethanol and water are mixed, they form a network of hydrogen bonds, which causes water molecules to be absorbed between ethanol molecules. This co-uptake of water and alcohol occurred on the surface of TPU-PNIPAM. As a result, water molecules were adsorbed within the first 20 min of rapid ethanol uptake. This phenomenon has been observed in previous studies [37]. According to a study by DeJaco, R.F. et al., when the uptake capacity of alcohol reaches a moderate level, the co-uptake of water reaches its maximum level [16]. However, the co-uptake of water then decreases as the uptake capacity of alcohol further increases. As a result, the concentration of ethanol decreased gradually during 20–50 min due to the co-uptake of alcohol and water [14]. Between 50 and 70 min, the process of uptake was controlled by thermodynamics. At this stage, the ethanol molecules that had been adsorbed on the hydrophobic point of TPU-PNIPAM underwent rearrangement, which resulted in the discharge of co-adsorbed water molecules from the hydrogen bond network. This led to a rapid reduction in the ethanol concentration in the ethanol/water mixed solution. In the last 70–90 min, the uptake curve became flat once again. This was because the hydroxyl group of the ethanol molecule provided a bonding site for the water molecule again after rearrangement. As a result, the overall performance was that the selective uptake capacity decreased, and the uptake reached equilibrium.

It was observed that a higher selectivity of ethanol could be achieved in low-concentration ethanol/water mixed solutions, particularly in 10 wt% ethanol/water mixed solutions. However, when the concentration of the solution was increased to 20 wt%, more ethanol molecules adsorbed on TPU-PNIPAM, which created numerous bonding sites for water molecules, ultimately leading to a decrease in selectivity. The results of the experiments indicated that TPU-PNIPAM could selectively adsorb ethanol/water mixtures with varying concentrations. However, the intertwined hydrogen bond network present in ethanol/water mixed solutions could be affected by the concentration of alcohol, which in turn could impact the hydrogen bond network within the solution and the bonding site of water on the surface of TPU-PNIPAM. As a result, the uptake curve of TPU-PNIPAM for ethanol exhibited a characteristic trend of fast-slow-fast.

#### 3.3.4. The Effect of Temperature on the Uptake Performance of TPU-PNIPAM

**Figure 9:**
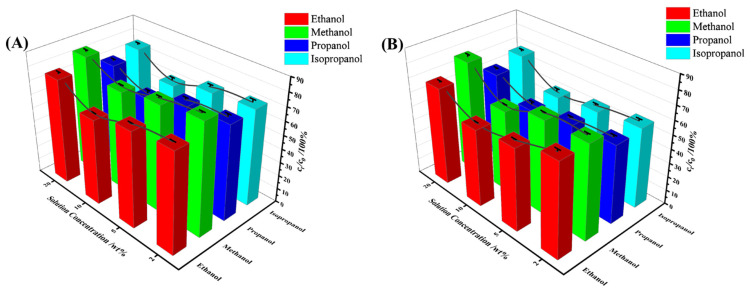
(**A**) The changes and the trends in solution concentration at room temperature. (**B**) The changes and the trends in solution concentration at 55 °C.

Various experiments were conducted using mixed ethanol and water solutions with different mass fractions (20 wt%, 10 wt%, 5 wt%, and 2 wt%) to examine how temperature affected the selective uptake performance of the TPU-PNIPAM copolymer for ethanol. Based on the blank control test conducted on a 20 wt% ethanol solution at 55 °C (Figure 9B), it was concluded that the influence of ethanol volatilization on the concentration change of the solution was negligible. Therefore, it could be inferred that the volatilization of 10 wt%, 5 wt%, and 2 wt% ethanol under the given heating condition had no significant impact on the concentration change of the solution. PNIPAM is a polymer that is sensitive to temperature. It was deemed that as the temperature increased, the hydrophobic alcohol molecules were more likely to be adsorbed and retained in the polymer network than the polar water molecules. This implied that warming provided PNIPAM with the ability to adsorb ethanol selectively. The uptake of alcohol molecules on PNIPAM promoted the dehydration of the polymer chain. This was because alcohol molecules preferentially adsorbed onto PNIPAM, which hindered hydrogen bonding between PNIPAM and water molecules. As a result, the hydrophobicity of the polymer chain was increased. This phenomenon has been observed in previous studies [38,39]. When the temperature was raised to 55 °C, the selective uptake performance of TPU-PNIPAM for mixed solutions of different concentrations of ethanol and water improved. This improvement was similar to the trend observed at room temperature, and the best ethanol selective uptake effect was observed at 10 wt%. As a result, the concentration of ethanol in the 10 wt% solution was decreased to 55.0% of its initial concentration. These observations are shown in Figure 8D. The experimental results demonstrate that by increasing temperature, TPU-PNIPAM could achieve better selective uptake capacity for ethanol in ethanol/water mixed solution.

#### 3.3.5. Effect of Different Alcohols on Selective Uptake Performance

**Figure A1:**
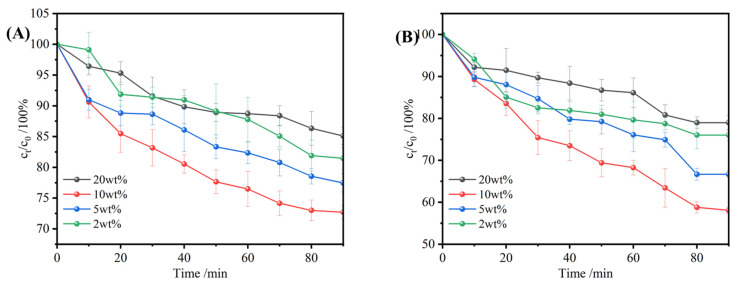
(**A**) Uptake effect of T-N1.5 on different concentrations of methanol at room temperature. (**B**) Uptake effect of T-N1.5 on different concentrations of methanol at 55 °C.

**Figure A2:**
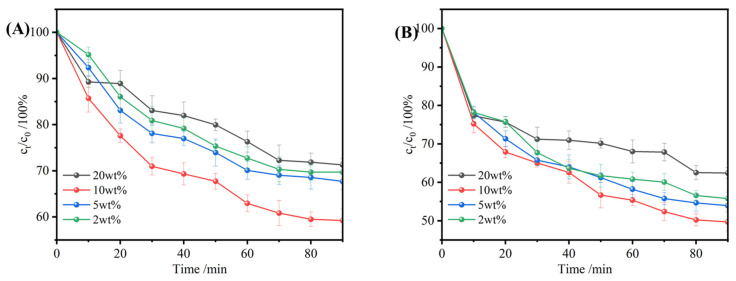
(**A**) Uptake effect of T-N1.5 on different concentrations of propanol at room temperature. (**B**) Uptake effect of T-N1.5 on different concentrations of propanol at 55 °C.

**Figure A3:**
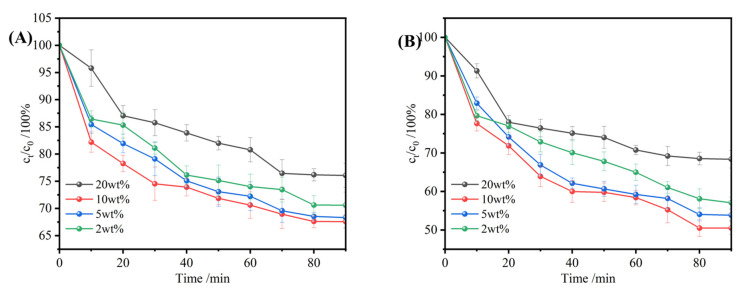
(**A**) Uptake effect of T-N1.5 on different concentrations of iso-propanol at room temperature. (**B**) Uptake effect of T-N1.5 on different concentrations of iso-propanol at 55 °C.

The study investigated the uptake properties of methanol, n-propyl alcohol, and isopropyl alcohol with different concentrations (20 wt%, 10 wt%, 5 wt%, and 2 wt%). Figure A1, Figure A2 and Figure A3 (which can be seen in Appendix A) display the copolymer’s selective uptake process of other alcohol–water mixtures under heating conditions and at room temperature, which followed similar rules as ethanol. TPU-PNIPAM showed similar selective uptake characteristics for all three alcohols, as indicated in Figure 9. TPU-PNIPAM demonstrated a selective capacity for adsorbing methanol/water, propanol/water, and isopropanol/water, similar to ethanol/water mixed solutions. TPU-PNIPAM exhibited the highest uptake capacity in a mixed solution of 10 wt% alcohol and water. Increasing the temperature to 55 °C further improved the selective uptake capacity of TPU-PNIPAM for alcohol. According to Figure 9, when subjected to the same conditions, TPU-PNIPAM exhibited varying degrees of selective uptake capacity for different alcohols. Methanol showed the least uptake, followed by ethanol, isopropyl alcohol, and propyl alcohol. For instance, after reaching an uptake equilibrium for a mixed solution with an initial concentration of 10 wt%, the solution concentrations dropped to 72.7%, 62.2%, 68.6%, and 58.2% (at room temperature) for methanol, ethanol, isopropyl alcohol, and propyl alcohol, respectively. At 55 °C, the solution concentrations dropped to 58.1%, 55.0%, 50.5%, and 48.7%, respectively. In a mixed solution of alcohol and water with an initial concentration of 20 wt%, the selective uptake differences between isopropyl alcohol and propyl alcohol were high, at 76.1%, 68.4%, 71.3%, and 62.4% at room temperature and 55 °C. This indicated that propanol was more preferentially adsorbed.

Short-chain alcohols like ethanol have properties similar to water and can easily form uniform mixed solutions with water. Therefore, to uptake alcohol from water, it is necessary to isolate or cover the hydrophilic point in the uptake material that can form hydrogen bonds with water. This will allow selective uptake of alcohol molecules using London dispersion interactions. In this paper, TPU and PNIPAM were combined through hydrogen bonds. This bonding method not only separated the hydrophilic hydrogen-bonding site on the surface of TPU, but it also occupied the same site within the PNIPAM network structure. Additionally, the exposed isopropyl group on the surface of PNIPAM was utilized as a hydrophobic point to disperse the alkyl groups of alcohol molecules. As the length of the alkyl chain in methanol, ethanol, and propanol increased, the strength of the London dispersion force between these alcohols and TPU-PNIPAM also increased. This meant that longer alkyl chains enhanced the selective uptake ability of the alcohols and improved the uptake capacity of mixed solutions, such as methanol/water, ethanol/water, and propanol/water. Regarding the difference in uptake properties of isopropyl alcohol and propyl alcohol in high-concentration alcohol/water mixed solutions, some researchers suggested that it is caused by the large steric hindrance of isopropyl alcohol [40]. We believe that the concentration of the alcohol and water mixture has a significant impact on the selective uptake performance, as mentioned earlier. Both factors work together to improve the uptake selectivity of propyl alcohol over isopropyl alcohol.

#### 3.3.6. Selective Uptake of Other Pollutants

**Figure 10:**
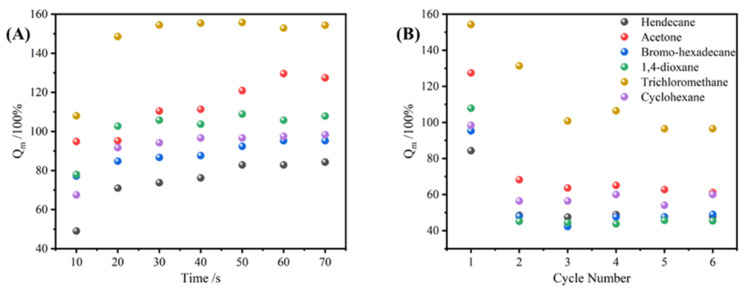
(**A**) The maximal uptake curve of T-N_1.5_ for trichloromethane, 1,4-dioxane, bromo-hexadecane, cyclohexane, acetone, and hendecane. (**B**) Repeated uptake properties of T-N_1.5_ for trichloromethane, 1,4-dioxane, bromo-hexadecane, cyclohexane, acetone, and hendecane.

To verify the universality of TPU-PNIPAM in the uptake of pollutants in water, this study tested the material with three organic solvents with densities greater than water (trichloromethane, 1,4-dioxane, and bromo-hexadecane) and three organic solvents with densities less than water (cyclohexane, acetone, and hendecane). Figure 10A shows that hydrophobic organic reagents reached uptake equilibrium more quickly than alcohols in the maximum uptake capacity test. Among these, TPU-PNIPAM demonstrated the highest maximum uptake performance for chloroform and acetone, achieving 154.28% and 127.39%, respectively.

**Figure 11:**
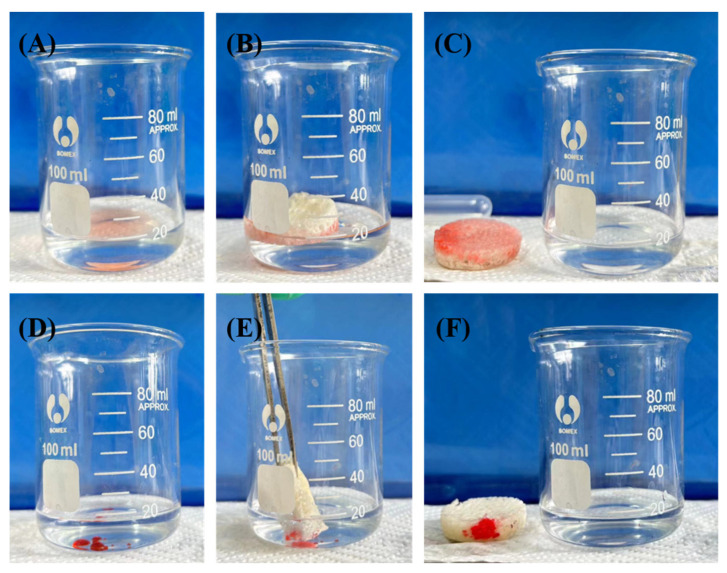
(**A**–**C**) Selective uptake of acetone by T-N_1.5_. (**D**–**F**) Selective uptake of trichloromethane by T-N_1.5_.

In testing the selective uptake performance of TPU-PNIPAM for organic solvents in aqueous solutions, trichloromethane and acetone were chosen to represent organic solvents with densities greater than water and less than water, respectively. For better visual differentiation between water and organic solvents, the organic solvents were stained with Sudan III. The experimental results depicted in Figure 11 demonstrate that TPU-PNIPAM could rapidly uptake organic solvents when they were nearby, regardless of whether they were submerged in water or floating on water. The above experiments demonstrated the universality of the selective uptake ability of TPU-PNIPAM for pollutants in aqueous solutions.

Most previously used PNIPAM copolymers for the selective separation of ethanol from aqueous solutions were prepared in the form of pervaporative membranes, demonstrating excellent ethanol and water separation [40,41]. By contrast, TPU-PNIPAM had a relatively weak ability to selectively absorb ethanol from aqueous solution. However, the preparation process of the TPU-PNIPAM copolymer was simple. In addition to ethanol, the TPU-PNIPAM copolymer had a certain selective adsorption effect on methanol, n-propanol, isopropanol, and other organic solvents in aqueous solutions. Its application scenarios were more extensive.

## 4. Conclusions

The TPU-PNIPAM copolymer was prepared by in situ polymerization and used to selectively extract ethanol from an aqueous solution. The molecular structures of TPU and PNIPAM both have hydrogen-bonding sites that can bind with water. By forming a hydrogen bond between the carbonyl oxygen and amino hydrogen, TPU and PNIPAM can expose the hydrophobic isopropyl group of PNIPAM to the greatest extent while covering the hydrophilic point. This resulted in the formation of a hydrophobic TPU-PNIPAM copolymer, which allowed for the selective uptake of alcohols in alcohol/water mixtures through London dispersion forces between alkyl groups. The performance of TPU-PNIPAM in selectively adsorbing alcohol/water mixed solutions was tested by comparing the concentration of alcohol/water before and after uptake. As the amount of PNIPAM loaded on the surface of TPU increased, the number of hydrogen bonds between TPU and PNIPAM increased, the hydrophilic point was largely covered, and the dispersion interaction between the exposed isopropyl group and the alcohol alkyl group was enhanced. This resulted in the TPU-PNIPAM having an enhanced selective uptake ability for alcohol/water mixed solutions. When PNIPAM loading reached a certain value, the alcohol in the aqueous solution could be completely removed by TPU-PNIPAM. When mixing alcohol and water solutions with varying initial concentrations, the uptake of alcohol molecules was accompanied by the co-uptake of water. The uptake curve of such mixtures showed a fast-slow-fast-equilibrium characteristic, with the two initial fast uptakes driven by kinetics and thermodynamics, respectively. As the amount of alcohol adsorbed reached a medium level, the co-uptake of water reached its maximum, leading to a gentle decrease in the uptake curve. When the temperature was raised to 55 °C, the TPU-PNIPAM exhibited better selective uptake performance on alcohol/water mixtures of varying concentrations. With an increase in temperature, alcohol molecules were more favorably adsorbed and retained in the polymer network than water molecules. This was because the hydrophobic isopropyl group of PNIPAM was more exposed, leading to enhanced van der Waals interactions with alcohol alkyl groups. In addition to the general uptake law, different types of alcohol/water mixed solutions exhibited varying degrees of selective uptake under the same conditions. The selective uptake capacity for different alcohols increased gradually in the order of methanol, ethanol, isopropyl alcohol, and propyl alcohol. This was due to the increased dispersion interactions of hydrophobic alkyl and alkyl alcohols. In the mixed solution of alcohol/water at 20 wt%, the steric hindrance of the alkyl group of isopropyl alcohol resulted in a less effective selective uptake effect compared to propyl alcohol. The TPU-PNIPAM copolymer was suitable for selectively adsorbing ethanol from aqueous solutions. When the PNIPAM load reached a certain value, TPU-PNIPAM was expected to completely remove the alcohol from the solution. In addition, TPU-PNIPAM exhibited selective adsorption for both denser and less dense organic solvents, which demonstrated the universality of TPU-PNIPAM in removing contaminants from aqueous solutions.

## Figures and Tables

**Table 1:** Relative areas of some characteristic peaks of TPU-PNIPAM in FT-IR and Raman spectra.

	T-N_0.5_	T-N_1.0_	T-N_1.5_
FT-IR -C(CH_3_)_2_	2.16%	2.93%	3.37%
Raman -NH_2_-	9.44%	8.93%	7.24%

## Data Availability

Data are contained within the article.

## Appendix A

The appendix lists the concentrations of methanol/water, propanol/water, and isopropanol/water mixtures over time.

## References

[1] Ramanathan K., Koch C.K., Oh S.H. (2012). Kinetic modeling of hydrocarbon adsorbers for gasoline and ethanol fuels. Chem. Eng. J..

[2] Bondy S.C., Guo S.X. (1994). Effect of Ethanol Treatment on Indices of Cumulative Oxidative Stress. Eur. J. Pharmacol..

[3] Faisal M., Khan S.B., Rahman M.M., Jamal A., Umar A. (2011). Ethanol Chemi-Sensor: Evaluation of Structural, Optical and Sensing Properties of CuO Nanosheets. Mater. Lett..

[4] Wang L., Huang H., Chang Y., Zhong C. (2021). Integrated High Water Affinity and Size Exclusion Effect on Robust Cu-Based Metal-Organic Framework for Efficient Ethanol—Water Separation. ACS Sustain. Chem. Eng..

[5] Lively R.P., Dose M.E., Thompson J.A., McCool B.A., Chance R.R., Koros W.J. (2011). Ethanol and water adsorption in methanol-derived ZIF-71. Chem. Commun..

[6] He X., Wang T., Huang J., Chen J., Li J. (2020). Fabrication and characterization of superhydrophobic PDMS composite membranes for efficient ethanol recovery via pervaporation. Sep. Purif. Technol..

[7] Liu Q., Huang B., Huang A. (2013). Polydopamine-based superhydrophobic membranes for biofuel recovery. J. Mater. Chem. A.

[8] Pan Y., Zhu T., Xia Q., Yu X., Wang Y. (2021). Constructing superhydrophobic ZIF-8 layer with bud-like surface morphology on PDMS composite membrane for highly efficient ethanol/water separation. J. Environ. Chem. Eng..

[9] Nalaparaju A., Zhao X., Jiang J. (2011). Biofuel purification by pervaporation and vapor permeation in metal-organic frameworks: A computational study. Energy Environ. Sci..

[10] Krishna R., van Baten J.M. (2020). Water/Alcohol Mixture Adsorption in Hydrophobic Materials: Enhanced Water Ingress Caused by Hydrogen Bonding. ACS Omega.

[11] Chovau S., Gaykawad S., Straathof AJ J., Van der Bruggen B. (2011). Influence of fermentation by-products on the purification of ethanol from water using pervaporation. Bioresour. Technol..

[12] Ahmed I., Pa N.F.C., Nawawi M.G.M., Rahman W.A.W.A. (2011). Modified Polydimethylsiloxane/Polystyrene Blended IPN Pervaporation Membrane for Ethanol/Water Separation. J. Appl. Polym. Sci..

[13] Xu C., Lu X., Wang Z. (2017). Effects of sodium ions on the separation performance of pure-silica MFI zeolite membranes. J. Membr. Sci..

[14] Kommu A., Singh J.K. (2017). Separation of Ethanol and Water Using Graphene and Hexagonal Boron Nitride Slit Pores: A Molecular Dynamics Study. J. Phys. Chem. C.

[15] Pitt W.W., Haag G.L., Lee D.D. (1983). Recovery of ethanol from fermentation broths using selective sorption-desorption. Biotechnol. Bioeng..

[16] DeJaco R.F., Loprete K., Pennisi K., Majumdar S., Siepmann J.I., Daoutidis P., Murnen H., Tsapatsis M. (2020). Modeling and simulation of gas separations with spiral-wound membranes. AIChE J..

[17] Cousin-Saint-Remi J., Denayer J.F.M. (2017). Applying the wave theory to fixed-bed dynamics of Metal-Organic Frameworks exhibiting stepped adsorption isotherms: Water/ethanol separation on ZIF-8. Chem. Eng. J..

[18] Nalaparaju A., Zhao X.S., Jiang J.W. (2010). Molecular Understanding for the Adsorption of Water and Alcohols in Hydrophilic and Hydrophobic Zeolitic Metal-Organic Frameworks. J. Phys. Chem. C.

[19] Delgado J.A., Agueda V.I., Uguina M.A., Sotelo J.L., Garcia A., Brea P., Garcia-Sanz A. (2013). Separation of ethanol-water liquid mixtures by on a polymeric resin Sepabeads 207^®^. Chem. Eng. J..

[20] de Lima G.F., Mavrandonakis A., de Abreu H.A., Duarte H.A., Heine T. (2013). Mechanism of Alcohol-Water Separation in Metal-Organic Frameworks. J. Phys. Chem. C.

[21] Liang F., Wang H., Liu G., Zhao J., Jin W. (2021). Designing highly selective and stable water transport channel through graphene oxide membranes functionalized with polyhedral oligomeric silsesquioxane for ethanol dehydration. J. Membr. Sci..

[22] Cao X., Zhou Y., Wei X., Zhai W., Zheng G., Dai K., Liu C., Shen C. (2018). Lightweight mechanical robust foam with a herringbone-like porous structure for oil/water separation and filtering. Polym. Testing.

[23] Petrović Z.S., Hong D., Javni I., Erina N., Zhang F., Ilavský J. (2013). Phase structure in segmented polyurethanes having fatty acid-based soft segments. Polymer..

[24] Wu T., Chen B. (2017). Facile Fabrication of Porous Conductive Thermoplastic Polyurethane Nanocomposite Films *via* Solution Casting. Sci. Rep..

[25] Muñoz-Chilito J., Lara-Ramos J.A., Marín L., Machuca-Martínez F., Correa-Aguirre J.P., Hidalgo-Salazar M.A., García-Navarro S., Roca-Blay L., Rodríguez L.A., Mosquera-Vargas E. (2023). Morphological Electrical and Hardness Characterization of Carbon Nanotube-Reinforced Thermoplastic Polyurethane (TPU) Nanocomposite Plates. Molecules.

[26] Karamikamkar S., Abidli A., Tafreshi O.A., Ghaffari-Mosanenzadeh S., Buahom P., Naguib H.E., Park C.B. (2024). Nanocomposite Aerogel Network Featuring High Surface Area and Superinsulation Properties. Chem. Mater..

[27] Liu C., Shi Y., Ye H., He J., Lin Y., Li Z., Lu J., Tang Y., Wang Y., Chen L. (2023). Functionalizing MXene with hypophosphite for highly fire safe thermoplastic polyurethane composites. Compos. Part A Appl. Sci. Manuf..

[28] Cong H.P., Qiu J.H., Yu S.H. (2015). Thermoresponsive Poly(*N*-isopropylacrylamide)/Graphene/Au Nanocomposite Hydrogel for Water Treatment by a Laser-Assisted Approach. Small.

[29] Backes S., Krause P., Tabaka W., Witt M.U., von Klitzing R. (2017). Combined Cononsolvency and Temperature Effects on Adsorbed PNIPAM Microgels. Langmuir..

[30] Wang N., Ru G., Wang L., Feng J. (2009). ^1^H MAS NMR Studies of the Phase Separation of Poly (*N*-isopropylacrylamide) Gel in Binary Solvents. Langmuir..

[31] Ou R., Wei J., Jiang L., Simon G.P., Wang H. (2016). Robust Thermoresponsive Polymer Composite Membrane with Switchable Superhydrophilicity and Superhydrophobicity for Efficient Oil-Water Separation. Environ. Sci. Technol..

[32] Shang J., Gao R., Su F., Wang H., Zhu D. (2020). Colloidal Probes of PNIPAM-Grafted SiO_2_ in Studying the Microrheology of Thermally Sensitive Microgel Suspensions. Adv. Polym. Technol..

[33] Wang M., Liu J., Yan K. (2023). Research on the performance and mechanism of asphalt modified by thermoplastic polyurethane with different chemical structures. Constr. Build. Mater..

[34] Rockwood D.N., Chase D.B., Akins R.E., Rabolt J.F. (2008). Characterization of electrospun poly(*N*-isopropyl acrylamide) fibers. Polymer.

[35] Hu W.-J., Li Y.-M., Li Y.-R., Wang D.-Y. (2023). Highly efficient intumescent flame retardant of dopamine-modified ammonium polyphosphate for the thermoplastic polyurethane elastomer. J. Therm. Anal. Calorim..

[36] Zeng K., Fang Y., Zheng S. (2009). Organic–inorganic hybrid hydrogels involving poly(*N*-isopropylacrylamide) and polyhedral oligomeric silsesquioxane: Preparation and rapid thermoresponsive properties. J. Polym. Sci. Part B Polym. Phys..

[37] Wang C.-H., Bai P., Siepmann J.I., Clark A.E. (2014). Deconstructing Hydrogen-Bond Networks in Confined Nanoporous Materials: Implications for Alcohol-Water Separation. Phys. Chem. C.

[38] Kojima H., Tanaka F. (2012). Reentrant volume phase transition of cross-linked poly(*N*-isopropylacrylamide) gels in mixed solvents of water/methanol. Soft Matter.

[39] Liu B., Wang J., Ru G., Liu C., Feng J. (2015). Phase Transition and Preferential Alcohol Adsorption of Poly(*N*,*N*-diethylacrylamide) Gel in Water/Alcohol Mixtures. Macromolecules.

[40] Ito Y., Ito T., Takaba H., Nakao S.-I. (2005). Development of gating membranes that are sensitive to the concentration of ethanol. J. Membr. Sci..

[41] Xie R., Song X.-L., Luo F., Liu Z., Wang W., Ju X.-J., Chu L.-Y. (2016). Ethanol-Responsive Poly(Vinylidene Difluoride) Membranes with Nanogels as Functional Gates. Chem. Eng. Technol..

